# *Coxiella burnetii* in the Arabian Peninsula: A One Health Perspective Through Systematic Review and Meta‐Analysis

**DOI:** 10.1155/tbed/8897201

**Published:** 2026-06-08

**Authors:** Abdallah F. M. Aldwekat, Md. Mazharul Islam, Mohammad Mahmudul Hassan, Muzzamil Atta, Mays M. H. Al-shehab, Hadi M. Yassine, Fatima Al-Khayat

**Affiliations:** ^1^ Department of Animal Resources, Ministry of Municipality, Doha, Qatar, bps.mme.gov.qa; ^2^ College of Arts and Sciences, Qatar University, Doha, Qatar, qu.edu.qa; ^3^ Faculty of Veterinary Medicine, Chattogram Veterinary and Animal Sciences University, Chattogram, 4225, Bangladesh, cvasu.ac.bd; ^4^ School of Veterinary Science, The University of Queensland, Gatton, Queensland, 4343, Australia, uq.edu.au; ^5^ Faculty of Pharmacy, Jadara University, Irbid, Jordan, jadara.edu.jo; ^6^ Biomedical Research Center, QU Health, Qatar University, Doha, Qatar, qu.edu.qa; ^7^ World Health Organization Collaborating Center for Research and Capacity Building on Emerging and Re-emerging Zoonotic Diseases, Qatar University, Doha, Qatar, qu.edu.qa

**Keywords:** animals, *Coxiella burnetii*, diversity, environment, humans, prevalence, risk factors

## Abstract

*Coxiella burnetii* is a zoonotic, obligate intracellular Gram‐negative bacterium that poses significant public and veterinary health challenges. This review aimed to assess the One Health burden of *C. burnetii* in the Arabian Peninsula by estimating the pooled prevalence and associated risk factors of the disease to guide control at the human–animal–environment interface. Following PRISMA guidelines and registration in OSF, a comprehensive literature search was conducted in the Arabic Collection Site, EBSCOhost, Embase, PubMed, Scopus, and Web of Science. A total of 320 articles were gathered, from where 64 articles were selected for the review. *C. burnetii* was reported in humans (28 studies), livestock (51), dogs (2), wildlife (3), and other samples (6). Human seroprevalence was estimated at 20.3%, with most cases attributed to exposure to ruminants. Among domestic ruminants, the pooled seroprevalence was 40.7%, highest in the Kingdom of Saudi Arabia (KSA; 72.2%), followed by the United Arab Emirates (UAE; 31.1%), and Iraq (19.5%). Large ruminants infested with ticks had a significantly higher seroprevalence (74.0%) compared to those without infestation (25.1%). Overall, 17.9% of samples tested from ruminants were positive by PCR. The highest detection rate was observed in reproductive samples (73.5%), followed by feces (61.2%) and milk (32.3%). Meta‐regression revealed significant associations between prevalence and specific factors, including country, tick infestation, and sample type. The review also suggests that additional, unmeasured, or context‐specific factors may influence disease dynamics. The study highlights substantial knowledge gaps despite the zoonotic and economic importance of *C. burnetii* in the region. A coordinated One Health surveillance framework is urgently needed to better understand its distribution, transmission dynamics, and impact on human and animal health and productivity in the Arabian Peninsula.

## 1. Introduction

*Coxiella burnetii* is a zoonotic, obligate intracellular Gram‐negative bacterium [1]. Once classified under *Rickettsia*, it is now placed in the order *Legionellales* due to its ability to grow within eukaryotic cells [2]. The organism exists in two morphologic forms: the large cell variant, which is intracellular, metabolically active and infectious, and the small cell variant, a spore‐like, extracellular, and environmentally resistant form [3]. The pathogen is highly infectious, and the inhalation of just 1–10 organisms can induce disease [4].

*C. burnetii* infects a wide range of vertebrate hosts as well as arthropods, especially ticks [1, 5]. In humans, infection manifests as Q fever, while in animals, it is known as coxiellosis, reflecting its public and veterinary health importance [6]. The sylvatic cycle of the pathogen is maintained through complex interactions among wildlife hosts, ticks, and the environment, independent of domestic livestock. Rodents, birds, and ticks play important roles in pathogen transmission within this cycle [7, 8]. Ticks facilitate the proliferation of *C. burnetii* within their midgut and gastric cells and subsequently excrete the bacterium in their feces and saliva [9]. Transmission at the human–animal interface occurs primarily through the inhalation of aerosols contaminated with animal birth products or dust [10]. Vertical transmission and unpasteurized dairy consumption are additional routes. Individuals in close contact with livestock are at high risk of infection [11]. Human Q fever is typically subclinical or mild, presenting a self‐limited flu‐like syndrome, and about 5% of cases can develop severe outcomes such as pneumonia, hepatitis, or chronic infections [12]. In animals, many infections remain asymptomatic, but reproductive disorders, including stillbirth, abortion, and infertility, are common [3, 4, 13].

The bacterium is globally distributed, including the Arabian Peninsula [14], which features deserts, plains, wadis, and mountain ranges [15, 16]. The Arabian Peninsula is globally significant for its petroleum resources, religious heritage, and a large expatriate population. Animal husbandry is a longstanding tradition in the region, with the Kingdom of Saudi Arabia (KSA) historically serving as a central hub for animal exchange. However, rapid economic development over recent decades has led to environmental degradation, primarily due to urbanization and overgrazing by livestock [17, 18]. Livestock production systems in this region are diverse, ranging from traditional smallholder and pastoral practices with mixed species, transboundary animal movement, and minimal biosecurity to increasingly large‐scale commercial dairy and poultry enterprises that follow intensive management and higher biosecurity standards [19, 20]. These factors, combined with ecological changes, climatic stress, and economic drivers, have fostered the emergence and re‐emergence of infectious diseases in the region [21–23].

*C*. *burnetii* has been detected in the Arabian Peninsula since 1968, initially in humans and subsequently in animals and environmental samples [24, 25]. Several molecular and serological studies have identified the pathogen in domestic and wild ruminants, carnivores, and environmental sources [24]. However, most studies are cross‐sectional and lack synthesis of prevalence and risk factors [26–31]. Consequently, the burden of coxiellosis in the human–animal environment in this region remains poorly understood. The objective of the systematic review and meta‐analysis was to consolidate the available evidence on *C. burnetii* in the Arabian Peninsula, estimating the pooled prevalence and identifying associated risk factors to inform regional disease control and prevention strategies.

## 2. Materials and Methods

This review was conducted in accordance with PRISMA guidelines [32] and a pre‐registered protocol on the Open Science Framework [33] to ensure transparent reporting, methodological rigor, and reproducible synthesis of the available evidence of *C. burnetii* in the Arabian Peninsula.

### 2.1. Data Search

**Figure 1:**
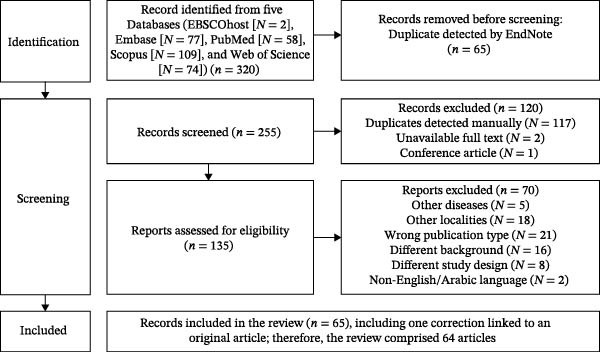
The PRISMA 2020 flow diagram describes the systematic review process for the selection of published articles, including the inclusion/exclusion criteria applied in the study.

A systematic search was conducted across six databases: Arabic Collection Site, EBSCOhost, Embase, PubMed, Scopus, and Web of Science (Figure 1). The search was conducted on December 31, 2024, without any restrictions on the publication date. The search terms were related to *Coxiella burnetii* and the countries of the Arabian Peninsula, limited to the title, abstract, and keywords fields. The retrieved records were exported into a single EndNote file (EndNote 21, Clarivate Analytics, Philadelphia, PA, USA), and duplicates were removed using the EndNote automatic tool. The records were subsequently screened in Rayyan (https://rayyan.qcri.org/) according to predefined inclusion and exclusion criteria [18].

The inclusion criteria encompassed cross‐sectional, longitudinal, and prevalence studies, case reports, and risk analyses conducted at any host level within the Arabian Peninsula. We excluded review and conference articles, book and book chapters, experimental studies, publications unrelated to *C. burnetii*, studies conducted outside the region, and papers in languages other than English or Arabic. The full texts of the eligible articles were retrieved from EndNote, PubMed, and ResearchGate. In the absence of articles from these databases, we procured them through the Qatar National Library Interlending and Document Supply Service. Additionally, we screened the reference lists of the included studies to identify any relevant publications that may have been missed during the initial systematic search.

### 2.2. Data Extraction

We extracted key variables, including sampling time, country of origin, animal characteristics (species, type, sex, age, tick infestation status, and reproductive disorder history), sample type (whole blood, serum, genital sample, and milk), sampling season, and other relevant study details.

### 2.3. Quality Assessment

We assessed the risk of bias and quality of the included studies using a modified critical appraisal tool for prevalence studies developed by Munn et al. [34]. This tool includes 10 questions to evaluate key bias domains, including confounding, selection bias, and measurement or analytical bias. Each question was rated as “yes,” “no,” “unclear,” or “not applicable” [35, 36]. For each study, a quality score was calculated as the proportion of “yes” responses among “yes,” “no,” and “unclear” responses, excluding “not applicable” items. Scores ranged from 0 to 100, and studies were categorized as low (<40), intermediate (≥40 to <70), and high (≥70) quality articles.

### 2.4. Data Analysis

The extracted data were compiled in Microsoft Excel spreadsheets (MS Office, 2019), and descriptive analyses, including article counts, percentages, and 95% confidence intervals (CIs), were conducted using R (RStudio, Version 4.3.1). Animals were classified into subgroups to assess risk factors: ruminants were grouped into large ruminants (cattle and camels), small ruminants (sheep and goats), and captive wild ruminants. The geographical distribution of coxiellosis was mapped using RStudio, showing positive host species (by serology or PCR) and the number of studies per country.

Pooled prevalence was estimated using a random‐effect model in RStudio, with both quantitative and subgroup meta‐analyses. Prevalence was categorized as estimated pooled seroprevalence (EPSP) for seropositive animals identified by ELISA or other antibody detection tests and estimated pooled molecular prevalence (EPMP) for PCR‐positive animals. For each pooled estimate, 95% CI and *p*‐values were calculated. Heterogeneity across studies was assessed using chi‐square (*χ* ^2^) analysis, followed by the *I* ^2^ statistic to quantify degree of heterogeneity between studies. Additionally, Tau‐squared (*τ* ^2^) test estimated between‐study variance in effect sizes. Study weights were assigned based on the relative contribution of each study’s sample size. Meta‐analysis results were presented using forest plots, while funnel plots were used to assess the potential for publication bias.

To further investigate the sources of heterogeneity and to assess the influence of specific factors on prevalence estimates, meta‐regression analyses were performed. These analyses were applied to the subgroups that showed significant heterogeneity in meta‐analysis. Prevalence data were modeled using a generalized linear mixed‐effects model with a logit transformation. The objective was to (i) quantify the contribution of selected factors to overall heterogeneity, (ii) identify significant predictors of prevalence, and (iii) explore potential unmeasured or context‐specific influences on disease distribution.

## 3. Results

### 3.1. Descriptive Statistics

The review included 64 published articles, the earliest dating back to 1994, which investigated *C. burnetii* in camels in the United Arab Emirates (UAE). Analysis of publication trends over 5‐year intervals revealed a steady increase in research output (Table 1). Most studies used immunological detection methods (*n* = 52, 81.25%), with a substantial number involving human subjects (*n* = 28, 43.75%). Among animal studies, small ruminants were the primary focus, with sheep and goats each accounted for 12 (18.75%) studies. Six studies (9.37%) investigated environmental sources, including soil, dust, molasses, ticks, and rodents. The majority (*n* = 55, 82.09%) of the articles used blood samples.

**Table 1:** Characteristics of the reviewed studies.

Factors	Number of articles, % (95% CI)	References
Publication years		
1991–1995	1, 1.56 (0.08–9.54)	[37]
1996–2000	2, 3.13 (0.54–11.81)	[28, 38]
2001–2005	2, 3.13 (0.54–11.81)	[39, 40]
2006–2010	9, 14.06 (7.02–25.53)	[29, 41–48]
2011–2015	12, 18.75 (10.47–30.85)	[49–60]
2016–2020	18, 28.13 (17.94–40.95)	[26, 30, 61–76]
2021–2024	21, 32.81 (21.90–45.80)	[27, 31, 77–95]
Coxiellosis tests methods		
Immunologic	52, 77.61 (65.48–86.53)	[26–29, 38–46, 48–56, 58–62, 64–74, 76, 78–80, 82–84, 87, 89–93, 95]
Molecular	24, 35.82 (24.74–48.53)	[29, 30, 46–48, 50, 57–60, 63, 71, 73, 75, 77, 80, 81, 84–89, 94, 95]
Other^a^	3, 4.47 (1.16–13.37)	[37, 38, 58]
Sampling sources/hosts		
Human	28, 41.79 (30.07–54.46)	[28, 38–44, 46, 48–50, 54–58, 61, 62, 65, 67–71, 83, 87, 92]
Sheep	12, 17.91 (9.98–29.59)	[26–28, 39, 59, 64, 66, 75, 76, 79, 84, 93]
Goat	12, 17.91 (9.98–29.59)	[26–28, 39, 59, 64, 66, 75, 76, 78, 90, 93]
Cattle	11, 16.42 (8.87–27.90)	[26, 59, 64, 66, 72–74, 77, 82, 85]
Camel	11, 16.42 (8.87–27.90)	[37, 45, 59, 60, 63, 66, 80, 86, 91, 93, 94]
Dog	2, 2.99 (0.52–11.31)	[51, 95]
Captive wild ruminant	3, 4.47 (1.16–13.37)	[29, 52, 53]
Others^b^	6, 8.96 (3.69–19.11)	[30, 31, 47, 81, 88, 89, 94]
Sample type		
Blood	55, 82.09 (70.41–90.01)	[26–29, 31, 37–46, 48–57, 59–63, 65–77, 79, 80, 82–84, 86, 88, 89, 91–95]
Urine	2, 2.99 (0.52–11.31)	[59, 60]
Feces	4, 5.97 (1.93–15.35)	[59, 60, 75, 84]
Milk	8, 11.94 (5.66–22.72)	[59, 60, 64, 73, 74, 78, 84, 90]
Genital	3, 4.47 (1.16–13.37)	[29, 84, 94]
Others^b^	4, 5.97 (1.93–15.35)	[30, 47, 81, 89]
Quality assessment		
High	41, 93.18 (80.29–98.22)	[26–29, 37, 39, 43–45, 49, 50, 52–55, 59, 60, 63–66, 68, 69, 72, 74–80, 82–86, 90–94]
Intermediate	3, 6.81 (1.78–19.71)	[40, 61, 73]

^a^Complement fixation test, isolation, et cetera.

^b^Soil, dust, rodent, molasses, ticks, et cetera.

Out of 64 articles, 44 (68.75%, 95% CI: 55.80–79.43) were eligible for meta‐analysis. In the quality assessment, most were rated high quality (*n* = 41, 93.18%), while 3 articles (*n* = 3, 6.81%) were of intermediate quality. None of them were classified as low quality. Meta‐analysis indicated high heterogeneity among the studies, with *I* ^2^ values exceeding 90% and *p*‐values <0.01. Funnel plots revealed potential publication bias, as evidenced by the asymmetric distribution of prevalence estimates.

### 3.2. Distribution of the Disease in the Arabian Peninsula

**Figure 2:**
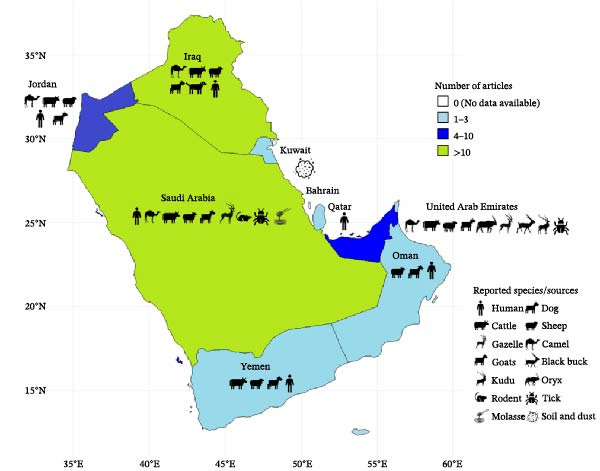
The countries of the Arabian Peninsula, where coxiellosis was reported at the human–animal–environment interface.

Geographically, most studies were conducted in Iraq (*n* = 18, 28.13%, 95% CI: 17.94–40.95) and the KSA (*n* = 15, 23.44%, 95% CI: 14.12–35.98) (Figure 2). Limited data were available from Kuwait, Qatar, Oman and Yemen, and no publications were found from Bahrain. Human Q fever cases were documented in Iraq, Jordan, KSA, Qatar, Oman, and Yemen. In animals, infections were reported in livestock ruminants (cattle, sheep, goats, and camels) across most countries. Cases involving dogs were reported in Iraq [51, 95], and rodents tested positive in KSA [31, 88]. Captive wild ruminants were also infected in KSA [53] and the UAE [29, 52], highlighting broader host involvement. Positive ticks were reported in the UAE [30] and KSA [94]. Environmental detection was also noted in Kuwait [47] and KSA [81].

### 3.3. Q Fever in Humans

**Figure 3:**
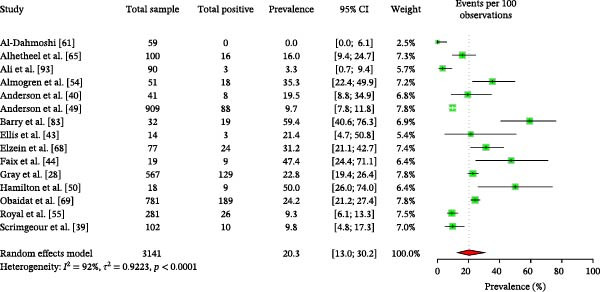
The estimated pooled seroprevalence of Q fever in humans in the Arabian Peninsula (the center dot represents point estimates and green squares represent the weight of each study to the meta‐analysis) [28, 39, 40, 43, 44, 49, 50, 54, 55, 61, 65, 68, 69, 83, 92].

The EPSP in suspected humans was 20.3% (Figure 3), with higher prevalence in KSA (33.4%) and Iraq (14.9%). Subgroup analysis by country or sex showed no significant differences (Table 2).

**Table 2:** Risk factors associated with Q fever in humans based on seroprevalence.

Sl. no.	Factors		Number of articles studied	Estimated pooled seroprevalence, 95% CI	Heterogeneity (*I* ^2^ (%)	*p*‐Value
1	Country	Iraq	8	14.9 (6.5–30.5)	94	*0.16*
		Kingdom of Saudi Arabia	4	33.4 (18.4–52.7)		
		Others	4	11.0 (3.5–29.9)		
2	Sex	Female	5	14.1 (6.7–27.2)	89	*0.69*
		Male	8	21.9 (11.0–38.8)		

The review identified clinical features and potential risk factors that could not be quantitatively assessed due to limited or inconsistent reporting. Most cases presented with respiratory symptoms [40, 43, 44, 49, 50, 55, 61, 92], while others involved fever of unknown origin and endocarditis [54, 65]. Many patients reported prior animal contact, including sheep, goats, cattle, and camels [28, 39, 40, 44, 49, 55, 68, 69]. Some individuals had a history of tick bites [40, 44, 55], consumption of unpasteurized milk or uncooked meat [28, 39, 40, 68, 69], rural residency [28, 43, 44, 69], and dust exposure [39, 40, 43, 44, 50, 69, 87].

### 3.4. Coxiellosis in Ruminants

#### 3.4.1. Seroprevalence and Associated Risk Factors

**Figure 4:**
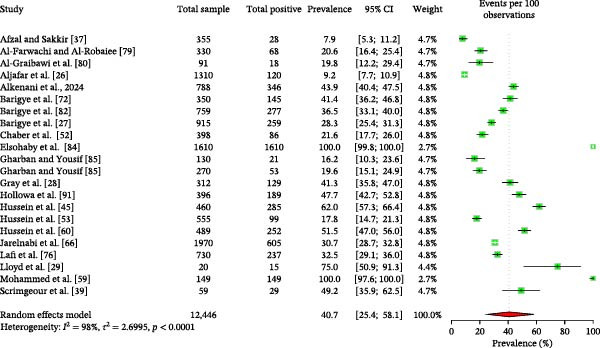
The estimated pooled seroprevalence of coxiellosis in livestock ruminants in the Arabian Peninsula (the center dot represents point estimates, and gray squares represent the weight of each study to the meta‐analysis) [26–29, 37, 39, 45, 52, 53, 59, 60, 66, 72–74, 76, 79, 80, 82, 84, 91, 93].

The EPSP of coxiellosis in ruminants across the Arabian Peninsula was 40.7% (Figure 4). When stratified by risk factors (Tables 3–6), the highest seroprevalence was observed in KSA at 72.2%, followed by the UAE at 31.1% and Iraq at 19.5% (Table 3). Among large ruminants, tick infestation emerged as a significant factor (*p* < 0.01) (Table 4). Infested animals had nearly a threefold higher seroprevalence (74.0%) than noninfected animals (25.1%) (Table 4). In camels, seroprevalence in KSA was significantly higher (*p* = 0.03) (54.8%) than other countries (21.3%) (Table 6).

**Table 3:** Estimated pooled seroprevalence of coxiellosis in livestock ruminants based on different risk factors in the Arabian Peninsula.

Sl. no.	Factor	Conditions	Number of articles	Estimated pooled seroprevalence, 95% CI	Heterogeneity (*I^2^* (%)	*p*‐Value
1	Ruminant type	Large ruminant	14	32.7 (20.1–48.5)	98	*0.26*
		Small ruminant	10	58.7 (19.7–89.2)		
2	Species	Camel	8	48.4 (23.8–73.8)	97	*0.38*
		Cattle	8	25.6 (14.8–40.6)		
		Goat	8	45 (29.2–61.8)		
		Sheep	10	48.8 (13.1–85.8)		
		Captive wild^a^	3	34.5 (9.9–71.7)		
3	Country	Iraq	4	19.5 (17.0–22.4)	98	*0.01*
		Kingdom of Saudi Arabia	8	72.2 (19.7–96.5)		
		United Arab Emirates	6	31.1 (15.8–51.9)		
		Others^b^	3	40.2 (31.7–49.4)		
4	Age	Adult	10	44.7 (15.5–78.1)	98	*0.12*
		Young	6	16.4 (6.8–34.6)		
5	Sex	Female	13	39.0 (18.5–64.4)	97	*0.25*
		Male	7	23.3 (12.6–39.0)		
6	History of abortion	Present	5	75.6 (12.6–98.5)	95	*0.31*
		Absent	3	35.7 (12.6–68.2)		

^a^Captive wild includes Gazelle and Oryx.

^b^Others include Jordan, Yemen, and Oman.

**Table 4:** Estimated pooled seroprevalence of coxiellosis in large ruminants based on different risk factors in the Arabian Peninsula.

Sl. no.	Factor	Conditions	Number of articles	Estimated pooled prevalence, 95% CI	Heterogeneity *I* ^2^ (%)	*p*‐Value
1	Country	Iraq	3	18.8 (15.6–22.5)	99	*0.10*
		Kingdom of Saudi Arabia	6	55.7 (20.6–85.9)		
		United Arab Emirates	3	24.8 (8.2–54.9)		
2	Age	Adult	7	31.6 (17.9–49.5)	97	*0.28*
		Young	4	17.3 (5.6–49.5)		
3	Sex	Female	9	32.3 (20.9–46.3)	97	*0.41*
		Male	3	12.7 (0.9–69.3)		
4	Tick infestation	No	3	25.1 (13.9–41.0)	94	*0.01*
		Yes	3	74.0 (64.9–81.3)		

**Table 5:** Estimated pooled seroprevalence of coxiellosis in livestock small ruminants based on different risk factors in the Arabian Peninsula.

Sl no.	Conditions	Risk factors	Number of articles	Estimated pooled prevalence, 95% CI	Heterogeneity *I* ^2^ (%)	*p*‐Value
1	Country	Kingdom of Saudi Arabia	5	82 (9.5–99.5)	97	0.28
		Others^a^	5	36.1 (24.1–50.1)		
2	Sex	Female	5	53.3 (2.9–97.7)	96	*0.33*
		Male	3	15.4 (10.1–22.7)		

^a^Others include Iraq, Jordan, Yemen, Oman, and United Arab Emirates.

**Table 6:** Estimated pooled seroprevalence of coxiellosis in different ruminant species based on different risk factors in the Arabian Peninsula.

Sl. no.	Conditions	Risk factors	Number of articles	Estimated pooled prevalence, 95% CI	Heterogeneity *I* ^2^ (%)	*p*‐Value
1	Country_Cattle	Kingdom of Saudi Arabia	3	38.5 (4.5–89.3)	96	*0.60*
		Others^a^	5	23.5 (13.9–37.0)		
2	Country_Camel	Kingdom of Saudi Arabia	5	54.8 (49.0–60.4)	97	*0.03*
		Others^b^	3	21.3 (6.5–51.2)		
3	Country_Sheep	Kingdom of Saudi Arabia	4	63.3 (1.5–99.5)	97	*0.66*
		Others^c^	5	37.5 (24.2–52.9)		
4	Country_Goat	Kingdom of Saudi Arabia	3	29.8 (15.4–49.8)	95	*0.14*
		Others^d^	3	48.3 (33.6–63.3)		

^a^Others include Iraq, Yemen, and United Arab Emirates.

^b^Others include Iraq, Jordan, and United Arab Emirates.

^c^Others include Iraq, Jordan, Yemen, and Oman.

^d^Others include Jordan, Yemen, Oman, and United Arab Emirates.

#### 3.4.2. Molecular Prevalence and Associated Risk Factors

**Figure 5:**
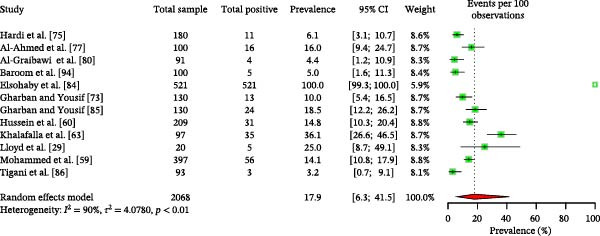
The estimated pooled molecular prevalence of *Coxiella burnetii* in ruminants in the Arabian Peninsula (the center dot represents point estimates and gray squares represent the weight of each study to the meta‐analysis) [29, 59, 60, 63, 73, 75, 77, 80, 84–86, 94].

Overall, 17.9% of tested samples were positive for *C. burnetii* by molecular methods (Figure 5). The highest detection rate occurred in reproductive samples (73.5%), including vaginal swabs and reproductive materials, followed by fecal (61.2%) and milk (32.3%) samples (Table 7).

**Table 7:** Estimated pooled molecular prevalence of coxiellosis in livestock ruminants based on different risk factors in the Arabian Peninsula.

Sl. no.	Factor	Conditions	Number of articles	Estimated pooled prevalence, 95% CI	Heterogeneity *I* ^2^ (%)	*p*‐Value
1	Ruminant type	Large ruminants	9	12.2 (7.4–19.6)	90	*0.42*
		Small ruminants	3	62.9 (0.4–19.6)		
2	Sample type	Whole blood/plasma/serum	7	7.0 (3.5–13.5)	89	*0.01*
		Reproductive samples	5	73.5 (10.2–98.6)		
		Fecal samples	4	61.2 (7.9–96.7)		
		Urine samples	2	23.3 (2.8–88.7)		
		Milk	5	32.3 (2.8–88.7)		
3	Species	Camel	6	10.4 (4.6–21.8)	87	*0.60*
		Cattle	4	15.8 (11.8–20.8)		
		Goat	2	10.1 (3.4–26.2)		
		Sheep	3	51.6 (0.1–99.9)		
4	Country	Iraq	5	10.4 (6.2–17.0)	90	*0.50*
		Kingdom of Saudi Arabia	4	44.9 (3.1–95.4)		
		United Arab Emirates	2	9.7 (1.1–50.6)		

#### 3.4.3. Meta‐Regression

Meta‐regression revealed no significant differences in ruminant seroprevalence by country (QM, *p* = 0.21), although KSA documented borderline higher prevalence than Iraq (*β* = 2.52) (Table 8). In large ruminants, tick infestation was associated with significantly higher seroprevalence (*β* = 3.46), confirming it as an important risk factor. In camels, country was a significant factor as KSA exhibited higher seroprevalence than the other countries (*β* = −2.29). PCR detection in ruminants varied by sample type: fecal (*β* = 4.09) and genital samples (*β* = 4.82) showed higher detection than blood, whereas milk and urine samples were not significantly different. Residual heterogeneity remained high across all models (*I* ^2^ = 90%–99%). This indicates substantial variability between studies, suggesting that other unmeasured or context‐specific factors likely influence disease dynamics.

**Table 8:** Summary of meta‐regression results assessing the effect of key factors on the prevalence of coxiellosis in animal populations in the Arabian Peninsula.

Outcome/population	Factors	Comparison	Logit (*β* ± SE)	95% CI	*p*‐Value
Seroprevalence in ruminants	Country	Kingdom of Saudi Arabia vs. Iraq	2.52 ± 1.30	−0.03–5.07	0.05
		Other countries^a^ vs. Iraq	1.07 ± 1.60	−2.07–4.22	0.50
		United Arab Emirates vs. Iraq	0.71 ± 1.36	−1.96–3.37	0.60
Seroprevalence in large ruminants	Tick	Present vs. absent	3.46 ± 1.17	1.16–5.77	<0.001
Seroprevalence in camels	Country	Other countries^b^ vs. Kingdom of Saudi Arabia	−2.29 ± 1.08	−4.41–0.17	0.03
PCR detection in ruminants	Sample	Feces vs. blood	4.09 ± 1.88	0.41–7.78	0.03
		Genital vs. blood	4.82 ± 1.80	1.29–8.35	0.01
		Milk vs. blood	2.77 ± 1.75	−0.65–6.19	0.11
		Urine vs. blood	2.06 ± 2.36	−2.56–6.68	0.38

^a^Others include Jordan and Yemen.

^b^Others include Iraq, Jordan, and United Arab Emirates.

#### 3.4.4. Additional Risk Factors

Reviewed articles checked several potential risk factors for coxiellosis in livestock ruminants. However, data limitations precluded comprehensive meta‐analysis for these associations. Serological studies indicated that multispecies farming and close interspecies contact were significantly associated with higher seropositivity [73, 74, 82, 91]. In camels, certain breeds showed higher prevalence, such as Magahim (44.64%) and Maghater (55.06%), compared to mixed breeds (50.83%) [60]. Among sheep, Blackhead breed had the highest prevalence (32.20%), followed by Droper (7.33%), Swaken (9.17%), and Awassi (0.27%) [26]. In goats, Omani breeds showed higher seroprevalence (52.94%) than Ardi (4.5%) and Somali breeds (34.88%) [26]. Sheep from semi‐nomadic farms were more frequently positive (72%) than those from stationary (40%) systems [76]. In cattle, infection rates were higher in larger herds [73, 74]. Crossbred animals showed higher prevalence than local or pure breeds in both serological and PCR‐based studies [73, 74, 85]. In addition, PCR‐based studies in camels showed higher positivity particularly those with reproductive disorders (78.6%) than other symptoms (21.45%) [63, 86, 94].

### 3.5. Coxiellosis in Other Animals and Samples

*C*. *burnetii* has been detected in various captive wild ruminants in the Arabian Peninsula, particularly in KSA and the UAE. Reported seroprevalence included Arabian oryx (*Oryx leucoryx*, 19.55%), sand gazelle (*Gazella leptoceros*, 7.33%), blackbuck (*Antilope cervicapra*, 5.56%), Speke’s gazelle (*Gazella spekei*, 8.57%), Dorcas gazelle (*Gazella Dorcas*, 33.33%), Mountain gazelle (*Gazella gazella*, 16.09%), Grant’s gazelle (*Nanger granti*, 6.67%), and lesser kudu (*Tragelaphus imberbis*, 5.00%) (Table 3) [29, 52, 53].

In domestic and synanthropic animals, infection was reported in dogs in Iraq (16.86%) [95]. Environmental contamination was confirmed in dust and soil samples from Kuwait (46.67%) [47]. Rodents were assessed in two studies: all samples tested negative in Qatar [89], whereas 16.5% were positive in KSA [31, 88].

*C. burnetii* DNA was also detected in molasses packets in KSA (5.5%) using 16S rRNA gene sequencing, suggesting potential contamination of consumable goods [81]. Ticks collected in KSA detected *C. burnetii* DNA (11.67%) [94]. In the UAE, 486 tick samples revealed *Coxiella*‐like endosymbionts using the *Icd* gene, but none were positive for *com1* gene and *IS1111* genes [30].

## 4. Discussion

This study provides an updated overview of coxiellosis at the human–animal–environment interface in the Arabian Peninsula. The diversity of hosts and sources highlights the wide regional distribution of the pathogen. Despite its clear zoonotic and economic importance, research activity varies across countries, possibly reflecting differences in national research priorities, surveillance policies, and reporting practices [96, 97]. Nevertheless, the steady rise in publications suggests increasing awareness and gradual improvement in the research environment.

*C. burnetii* was first confirmed in humans in the Arabian Peninsula in 1968 [24, 98]. In our study, the EPSP in suspected humans (20.3%) was lower than that reported for the WHO EMRO region (25.5%), which includes the Arabian Peninsula. It was also lower than the prevalence estimates from Iran (27%) and Egypt (25.6%) [99, 100] but higher than those from South Asia (9.2%) and Australia (5.6%) [101, 102]. In contrast, the EPSP in suspected ruminants (40.7%) in this study exceeded averages for the WHO EMRO region (22.4%), Iran (27%), and South Asia (11.9%) [99, 100, 102]. Similarly, the EPMP in ruminants (17.9%) was higher than that in Iran (5%) and South Asia (5.3%) [100, 102].

Although human seroprevalence did not differ among countries, notable variations occurred in ruminants. Camels in KSA exhibited higher seroprevalence than those in neighboring countries. This likely reflects Saudi Arabia’s vast landscape, larger camel population, and extensive animal trade and movement, which facilitate interspecies contact and pathogen circulation. Stronger surveillance and diagnostic capacity may also contribute to a higher reported prevalence compared with countries having smaller camel populations and weaker research infrastructure. Previous studies show that Q fever prevalence can vary not only between countries but also within regions and cities [99, 100, 102].

Ticks are recognized vectors in maintaining the sylvatic cycle of *C. burnetii* [9]. In KSA, tick infestation in ruminants has been reported at 27.65% [103]. In this study, tick infestation was a significant factor for seroprevalence, consistent with the findings from South Asia [102]. Molecular prevalence was highest in reproductive samples, supporting evidence that infected animals shed large quantities of *C. burnetii* during abortion or parturition [104–106].

Meta‐regression indicated that country, tick infestation, and sample type alone did not fully explain serological or molecular prevalence, suggesting additional interacting factors. Local contexts, including poor biosecurity in nomadic farms, multispecies farming, close human–animal contact, raw milk consumption, and cross‐border animal movement without quarantine, likely influence disease distribution [107]. However, limited research restricts a comprehensive understanding of One Health transmission pathways in the region, highlighting the need for further epidemiological studies.

Despite its zoonotic potential, considerable knowledge gaps remain within the One Health framework. Given the pathogen’s ability to infect birds and multiple animal species, including humans, and persist in the environment, prevention and control require an integrated approach across human, animal, and environmental sectors. Strengthened surveillance is needed to map distribution, assess pathogen diversity, and quantify health and economic burdens [108]. These efforts are constrained by limited research infrastructure, enabling environments, and political or administrative commitment. Prolonged instability in parts of Yemen, Iraq, and Jordan may further weaken surveillance and public health responses to coxiellosis and other zoonoses [109, 110]. Cultural barriers may also limit the adoption of evidence‐based interventions [107]. Addressing these challenges requires collaboration among medical and veterinary professionals, environmental scientists, community leaders, policymakers, media, and law enforcement authorities to develop coordinated One Health strategies [111].

Given shared cultural practices and active livestock trade across the region, inter‐country collaboration is essential for controlling zoonoses, such as Q fever. Regional bodies such as the Gulf Centre for Disease Prevention and Control can play a pivotal role in coordinating joint initiatives, facilitating research, and supporting effective prevention and control measures.

## 5. Conclusions

*C. burnetii* is widely prevalent among humans, animals, and environmental sources in the Arabian Peninsula. Infection has been documented in humans, domestic and wild ruminants, and pets, while the pathogen has been detected in environmental samples, food products, and ticks. Seroprevalence in humans and ruminants appear higher than in many neighboring regions, highlighting the need for coordinated control strategies. Besides geographical location, tick infestation and reproductive tissues or samples were significant risk factors. Despite the recognized zoonotic and economic importance of coxiellosis, substantial knowledge gaps remain, with several countries lacking published data and others having limited information. A comprehensive One Health surveillance approach is essential to clarify the disease burden, distribution, diversity, and risk factors, as well as impacts on human and animal health and productivity. Strengthened regional cooperation is therefore recommended to facilitate knowledge exchange, build technical capacity, and share laboratory resources to support effective prevention and control.

## Author Contributions

Conceptualization: Abdallah F. M. Aldwekat, Md. Mazharul Islam, Hadi M. Yassine, and Fatima Al‐Khayat. Methodology: Abdallah F. M. Aldwekat, Md. Mazharul Islam, and Mays M. H. Al‐shehab. Formal analysis, writing – original draft preparation: Abdallah F. M. Aldwekat and Md. Mazharul Islam. Writing – review and editing: Abdallah F. M. Aldwekat, Md. Mazharul Islam, Mays M. H. Al‐shehab, Muzzamil Atta, Mohammad Mahmudul Hassan, Hadi M. Yassine, and Fatima Al‐Khayat.

## Funding

This review was funded by the Biomedical Research Center, QU Health, Qatar University, Doha, Qatar under Project Number MME03‐1128‐210032.

## Disclosure

All authors have read and agreed to the final version of the manuscript.

## Conflicts of Interest

The authors declare no conflicts of interest.

## Data Availability

The work was conducted using publicly available data. There are no additional data to make open to the readers.

## References

[1] Aldwekat A. F. M. , Lorestani N. , and Shabani F. , Impacts of Climate Change on the Global Spread and Habitat Suitability of *Coxiella burnetii*: Future Projections and Public Health Implications, The Journal of Climate Change and Health. (2025) 22, 10.1016/j.joclim.2025.100442, 100442. 41646248 PMC12851218

[2] Derrick E. , “Q” Fever, a New Fever Entity: Clinical Features, Diagnosis and Laboratory Investigation, 1937, 281–299. 10.1093/clinids/5.4.790 6622891

[3] Maurin M. and Raoult D. , Q Fever, Clinical Microbiology Reviews. (1999) 12, no. 4, 518–553, 10.1128/CMR.12.4.518. 10515901 PMC88923

[4] Lang G. H. , Coxiellosis ^∗^(Q Fever) in Animals, Q Fever, 2024, I, CRC Press, 23–48.

[5] Ebani V. V. , *Coxiella burnetii* Infection in Cats, Pathogens. (2023) 12, no. 12, 1415. 38133298 10.3390/pathogens12121415 PMC10747756

[6] Robi D. T. , Demissie W. , and Temteme S. , Coxiellosis in Livestock: Epidemiology, Public Health Significance, and Prevalence of *Coxiella burnetii* Infection in Ethiopia, Veterinary Medicine: Research and Reports. (2023) 14, 145–158, 10.2147/VMRR.S418346. PMC10443632 37614223

[7] Körner S. , Makert G. R. , Ulbert S. , Pfeffer M. , and Mertens-Scholz K. , The Prevalence of *Coxiella burnetii* in Hard Ticks in Europe and Their Role in Q Fever Transmission Revisited-A Systematic Review, Frontiers in Veterinary Science. (2021) 8, 10.3389/fvets.2021.655715, 655715. 33981744 PMC8109271

[8] Celina S. S. and Cerný J. , *Coxiella burnetii* in Ticks, Livestock, Pets and Wildlife: A Mini-Review, Frontiers in Veterinary Science. (2022) 9, 10.3389/fvets.2022.1068129, 1068129. 36439350 PMC9691889

[9] Duron O. , Noël V. , and McCoy K. D. , et al.The Recent Evolution of a Maternally-Inherited Endosymbiont of Ticks Led to the Emergence of the Q Fever Pathogen, *Coxiella burnetii* , PLoS Pathogens. (2015) 11, no. 5, 10.1371/journal.ppat.1004892, e1004892. 25978383 PMC4433120

[10] Vanderburg S. , Rubach M. P. , Halliday J. E. , Cleaveland S. , Reddy E. A. , and Crump J. A. , Epidemiology of *Coxiella burnetii* Infection in Africa: A OneHealth Systematic Review, PLoS Neglected Tropical Diseases. (2014) 8, no. 4, 10.1371/journal.pntd.0002787, e2787. 24722554 PMC3983093

[11] Porter S. R. , Czaplicki G. , Mainil J. , Guattéo R. , and Saegerman C. , Q Fever: Current State of Knowledge and Perspectives of Research of a Neglected Zoonosis, International Journal of Microbiology. (2011) 2011, no. 1, 10.1155/2011/248418, 248418. 22194752 PMC3238387

[12] Anderson A. , Bijlmer H. , and Fournier P. E. , et al.Diagnosis and Management of Q Fever—United States, 2013: Recommendations From CDC and the Q Fever Working Group, Mmwr Recommendations and Reports. (2013) 62, no. RR-03, 1–30. 23535757

[13] Rodolakis A. , Q Fever in Dairy Animals, Annals of the New York Academy of Sciences. (2009) 1166, no. 1, 90–93. 19538267 10.1111/j.1749-6632.2009.04511.x

[14] McDade J. E. , Historical Aspects of Q Fever, Q Fever, 2024, I, CRC Press, 5–21.

[15] Alrajhi K. , Bibi S. , and Abu-Dieyeh M. , Diversity, Distribution, and Applications of Arbuscular Mycorrhizal Fungi in the Arabian Peninsula, Saudi Journal of Biological Sciences. (2024) 31, no. 2, 10.1016/j.sjbs.2023.103911, 103911. 38268781 PMC10805673

[16] Ghazanfar S. A. , Biogeography and Conservation in the Arabian Peninsula: A Present Perspective, Plants. (2024) 13, no. 15, 2091. 39124209 10.3390/plants13152091 PMC11313995

[17] Al-Atiyat R. M. , Aljumaah R. S. , Alshaikh M. A. , and Abudabos A. M. , Microsatellite-Based Genetic Structure and Diversity of Local Arabian Sheep Breeds, Frontiers in Genetics. (2018) 9, 10.3389/fgene.2018.00408. PMC6167516 30319690

[18] Islam M. M. , Elfadl A. K. , and Naeem A. , et al.Epidemiology and Diversity of Paratuberculosis in the Arabian Peninsula: A Systematic Review and Meta-Analysis With Implications for One Health, Pathogens. (2025) 14, no. 9, 10.3390/pathogens14090841. PMC12472523 41011742

[19] Chaber A. L. and Saegerman C. , Biosecurity Measures Applied in the United Arab Emirates-A Comparative Study Between Livestock and Wildlife Sectors, Transboundary and Emerging Diseases. (2017) 64, no. 4, 1184–1190, 10.1111/tbed.12488. 26961479

[20] Elawad E. , Atta M. , Agied M. , Ahmed M. , Alyahri H. , and Elbashir M. , Livestock Practices: Traditional Animal Holdings Classification in Qatar 2020 Towards Sustainable Food Security, Journal of Advanced Zoology. (2023) 44, no. 4, 409–418, 10.17762/jaz.v44i4.1941.

[21] Jones B. A. , Grace D. , and Kock R. , et al.Zoonosis Emergence Linked to Agricultural Intensification and Environmental Change, Proceedings of the National Academy of Sciences of the United States of America. (2013) 110, no. 21, 8399–8404, 10.1073/pnas.1208059110. 23671097 PMC3666729

[22] Perveen N. , Muzaffar S. B. , and Al-Deeb M. A. , Ticks and Tick-Borne Diseases of Livestock in the Middle East and North Africa: A Review, Insects. (2021) 12, no. 1, 10.3390/insects12010083. PMC7835866 33477991

[23] Bett B. , Kiunga P. , and Gachohi J. , et al.Effects of Climate Change on the Occurrence and Distribution of Livestock Diseases, Preventive Veterinary Medicine. (2017) 137, no. Pt B, 119–129, 10.1016/j.prevetmed.2016.11.019. 28040271

[24] Devaux C. A. , Osman I. O. , Million M. , and Raoult D. , *Coxiella burnetii* in Dromedary Camels (*Camelus dromedarius*): A Possible Threat for Humans and Livestock in North Africa and the Near and Middle East?, Frontiers in Veterinary Science. (2020) 7, 10.3389/fvets.2020.558481, 558481. 33251255 PMC7674558

[25] Leski T. A. , Malanoski A. P. , Gregory M. J. , Lin B. , and Stenger D. A. , Application of a Broad-Range Resequencing Array for Detection of Pathogens in Desert Dust Samples From Kuwait and Iraq, Applied and Environmental Microbiology. (2011) 77, no. 13, 4285–4292, 10.1128/AEM.00021-11. 21571877 PMC3127696

[26] Aljafar A. , Salem M. , Housawi F. , Zaghawa A. , and Hegazy Y. , Seroprevalence and Risk Factors of Q-Fever (*C. burnetii* Infection) Among Ruminants Reared in the Eastern Region of the Kingdom of Saudi Arabia, Tropical Animal Health and Production. (2020) 52, no. 5, 2631–2638, 10.1007/s11250-020-02295-6. 32458350

[27] Barigye R. , Hassan N. A. D. , and Abdalla-Alfaki I. M. , et al.Pilot Serosurvey of *Coxiella burnetii* in Domesticated Small Ruminants in the United Arab Emirates, Tropical Animal Health and Production. (2022) 54, no. 3, 10.1007/s11250-022-03150-6. 35377029

[28] Gray G. C. , Kassira E. N. , and Rodier G. , et al.Remote Village Survey for Agents Causing Hepatosplenic Disease in the Republic of Yemen, Tropical Doctor. (1999) 29, no. 4, 212–219, 10.1177/004947559902900408. 10578634

[29] Lloyd C. , Stidworthy M. F. , and Ulrich W. , *Coxiella burnetii* Abortion in Captive Dama Gazelle (*Gazella Dama*) in the United Arab Emirates, Journal of Zoo and Wildlife Medicine. (2010) 41, no. 1, 83–89, 10.1638/2009-0005.1. 20722258

[30] Al-Deeb M. A. , Frangoulidis D. , and Walter M. C. , et al.Coxiella-Like Endosymbiont in Argasid Ticks (*Ornithodoros muesebecki*) From a Socotra Cormorant Colony in Umm Al Quwain, United Arab Emirates, Ticks and Tick-borne Diseases. (2016) 7, no. 1, 166–171, 10.1016/j.ttbdis.2015.10.012. 26515059

[31] Alotaibi B. H. , Amor N. , Merella P. , Mohammed O. B. , and Alagaili A. N. , Genetic Diversity of Wild Rodents and Detection of *Coxiella burnetii*, the Causative Agent of Q Fever, in Saudi Arabia, Veterinary Research Communications. (2022) 46, no. 3, 769–780, 10.1007/s11259-022-09897-5. 35132522

[32] PRISMA , PRISMA 2020 Statement Paper, 2025, https://www.prisma-statement.org/prisma-2020-statement.

[33] Aldwekat A. F. M. , Islam M. M. , Yassine H. , and Al-Khayat F. A. , Systemic Review and Meta-Analysis to Estimate the Current Situation of *Coxiella burnetii* at the One Health Interface in the Arabian Peninsula, 10.17605/OSF.IO/H73ZJ.

[34] Munn Z. , Moola S. , Riitano D. , and Lisy K. , The Development of a Critical Appraisal Tool for Use in Systematic Reviews Addressing Questions of Prevalence, International Journal of Health Policy and Management. (2014) 3, no. 3, 123–128, 10.15171/ijhpm.2014.71. 25197676 PMC4154549

[35] Islam M. M. , Farag E. , and Eltom K. , et al.Rodent Ectoparasites in the Middle East: A Systematic Review and Meta-Analysis, Pathogens. (2021) 10, no. 2, 10.3390/pathogens10020139. PMC7911898 33572506

[36] Islam M. M. , Khanom H. , and Farag E. , et al.Global Patterns of Middle East Respiratory Syndrome Coronavirus (MERS-CoV) Prevalence and Seroprevalence in Camels: A Systematic Review and Meta-Analysis, One Health. (2023) 16, 10.1016/j.onehlt.2023.100561, 100561. 37200564 PMC10166617

[37] Afzal M. and Sakkir M. , Survey of Antibodies against Various Infectious Disease Agents in Racing Camels in Abu Dhabi, United Arab Emirates, Revue Scientifique et Technique de l’OIE. (1994) 13, no. 3, 787–792, 10.20506/rst.13.3.794. 7949353

[38] Scrimgeour E. M. , Johnston W. J. , Al Dhahry S. H. S. , El-Khatim H. S. , John V. , and Musa M. , First Report of Q Fever in Oman, Emerging Infectious Diseases. (2000) 6, no. 1, 74–76, 10.3201/eid0601.000114. 10653575 PMC2627974

[39] Scrimgeour E. M. , Al-Ismaily S. I. , Rolain J. M. , Al-Dhahry S. H. , El-Khatim H. S. , and Raoult D. , Q Fever in Human and Livestock Populations in Oman, Annals of the New York Academy of Sciences. (2003) 990, no. 1, 221–225, 10.1111/j.1749-6632.2003.tb07366.x. 12860629

[40] Anderson A. D. , Smoak B. , Shuping E. , Ockenhouse C. , and Petruccelli B. , Q Fever and the US Military, Emerging Infectious Diseases. (2005) 11, no. 8, 1320–1322, 10.3201/eid1108.050314. 16110586 PMC3320491

[41] Gleeson T. D. , Decker C. F. , Johnson M. D. , Hartzell J. D. , and Mascola J. R. , Q Fever in US Military Returning From Iraq, The American Journal of Medicine. (2007) 120, no. 9, e11–e12, 10.1016/j.amjmed.2007.03.020. 17765028

[42] Hartzell J. D. , Peng S. W. , and Wood-Morris R. N. , et al.Atypical Q Fever in US Soldiers, Emerging Infectious Diseases. (2007) 13, no. 8, 1247–1249, 10.3201/eid1308.070218. 17953104 PMC2828091

[43] Ellis S. B. , Appenzeller G. , and Lee H. , et al.Outbreak of Sandfly Fever in Central Iraq, September 2007, Military Medicine. (2008) 173, no. 10, 949–953, 10.7205/MILMED.173.10.949. 19160611

[44] Faix D. J. , Harrison D. J. , and Riddle M. S. , et al.Outbreak of Q Fever among US Military in Western Iraq, June-July 2005, Clinical Infectious Diseases. (2008) 46, no. 7, e65–e68, 10.1086/528866. 18444807

[45] Hussein M. F. , Alshaikh M. , Gad El-Rab M. O. , Aljumaah R. S. , Gar El Nabi A. R. , and Abdel Bagi A. M. , Serological Prevalence of Q Fever and Chlamydiosis in Camels in Saudi Arabia, Journal of Animal and Veterinary Advances. (2008) 7, no. 6, 685–688.

[46] Ake J. A. , Massung R. F. , Whitman T. J. , and Gleeson T. D. , Difficulties in the Diagnosis and Management of a US Servicemember Presenting With Possible Chronic Q Fever, Journal of Infection. (2010) 60, no. 2, 175–177, 10.1016/j.jinf.2009.09.010. 19766138

[47] Leski T. A. , et al.Analysis of Dust Samples From the Middle East Using High Density Resequencing Microarray “RPM-TEI”, 7666, *Conference on Sensors, and Command, Control, Communications, and Intelligence (C31) Technologies for Homeland Security and Homeland Defense IX*, 2010, SPIE, 273–283.

[48] Miceli M. H. , Veryser A. K. , Anderson A. D. , Hofinger D. , Lee S. A. , and Tancik C. , A Case of Person-to-Person Transmission of Q Fever From an Active Duty Serviceman to his Spouse, Vector-Borne and Zoonotic Diseases. (2010) 10, no. 5, 539–541, 10.1089/vbz.2009.0101. 20020811

[49] Anderson A. D. , Baker T. R. , Littrell A. C. , Mott R. L. , Niebuhr D. W. , and Smoak B. L. , Seroepidemiologic Survey for *Coxiella burnetii* Among Hospitalized US Troops Deployed to Iraq, Zoonoses and Public Health. (2011) 58, no. 4, 276–283, 10.1111/j.1863-2378.2010.01347.x. 20880090

[50] Hamilton L. R. , George D. L. , Scoville S. L. , Hospenthal D. R. , and Griffith M. E. , PCR for Rapid Diagnosis of Acute Q Fever at a Combat Support Hospital in Iraq, Military Medicine. (2011) 176, no. 1, 103–105, 10.7205/MILMED-D-10-00111. 21305969

[51] Havas K. A. and Burkman K. , A Comparison of the Serological Evidence of *Coxiella burnetii* Exposure Between Military Working Dogs and Feral Canines in Iraq, Military Medicine. (2011) 176, no. 10, 1101–1103, 10.7205/MILMED-D-11-00025. 22128642

[52] Chaber A. L. , Lloyd C. , O’Donovan D. , McKeown S. , Wernery U. , and Bailey T. , A Serologic Survey for *Coxiella burnetii* in Semi-Wild Ungulates in the Emirate of Dubai, United Arab Emirates, Journal of Wildlife Diseases. (2012) 48, no. 1, 220–222, 10.7589/0090-3558-48.1.220. 22247396

[53] Hussein M. F. , Al-Khalifa I. M. , and Aljumaah R. S. , et al.Serological Prevalence of *Coxiella burnetii* in Captive Wild Ruminants in Saudi Arabia, Comparative Clinical Pathology. (2012) 21, no. 1, 33–38, 10.1007/s00580-010-1061-y.

[54] Almogren A. , Shakoor Z. , Hasanato R. , and Adam M. H. , Q Fever: A Neglected Zoonosis in Saudi Arabia, Annals of Saudi Medicine. (2013) 33, no. 5, 464–468, 10.5144/0256-4947.2013.464. 24188940 PMC6074889

[55] Royal J. , Riddle M. S. , Mohareb E. , Monteville M. R. , Porter C. K. , and Faix D. J. , Seroepidemiologic Survey for *Coxiella burnetii* Among US Military Personnel Deployed to Southwest and Central Asia in 2005, The American Society of Tropical Medicine and Hygiene. (2013) 89, no. 5, 991–995, 10.4269/ajtmh.12-0174. PMC3820350 24043692

[56] White B. , Brooks T. , and Seaton R. A. , Q Fever in Military and Paramilitary Personnel in Conflict Zones: Case Report and Review, Travel Medicine and Infectious Disease. (2013) 11, no. 2, 134–137, 10.1016/j.tmaid.2012.11.001. 23218785

[57] Angelakis E. , Johani S. , Ahsan A. , Memish Z. , and Raoult D. , Q Fever Endocarditis and New *Coxiella burnetii* Genotype, Saudi Arabia, Emerging Infectious Diseases. (2014) 20, no. 4, 726–728, 10.3201/eid2004.131603. 24655815 PMC3966385

[58] D’Amato F. , Robert C. , Azhar E. I. , Fournier P. E. , and Raoult D. , Draft Genome Sequence of *Coxiella burnetii* Strain Cb196, an Agent of Endocarditis in Saudi Arabia, Genome Announcements. (2014) 2, no. 6, 10–1128, 10.1128/genomeA.01180-14. PMC4246156 25428964

[59] Mohammed O. B. , Jarelnabi A. A. , and Aljumaah R. S. , et al. *Coxiella burnetii*, the Causative Agent of Q Fever in Saudi Arabia: Molecular Detection From Camel and Other Domestic Livestock, Asian Pacific Journal of Tropical Medicine. (2014) 7, no. 9, 715–719, 10.1016/S1995-7645(14)60122-X.

[60] Hussein M. F. , Alshaikh M. A. , Al-Jumaah R. S. , GarelNabi A. , Al-Khalifa I. , and Mohammed O. B. , The Arabian Camel (*Camelus dromedarius*) as a Major Reservoir of Q Fever in Saudi Arabia, Comparative Clinical Pathology. (2015) 24, no. 4, 887–892, 10.1007/s00580-014-2002-y.

[61] Al-Dahmoshi H. O. M. , Rapid Investigation of Uncultivable Respiratory Tract Bacteria Among Tuberculosis Patients in Hilla City, Iraq, Research Journal of Pharmaceutical, Biological and Chemical Sciences. (2016) 7, no. 6, 2723–2729.

[62] Robinson W. P. and Schuksz M. , Surgical and Antimicrobial Management of a Thoracic Aortic Aneurysm Due to Q Fever: A Case Report and Brief Review, Vascular and Endovascular Surgery. (2016) 50, no. 4, 290–294, 10.1177/1538574416642876. 27075992

[63] Khalafalla A. I. , Al Eknah M. M. , Abdelaziz M. , and Ghoneim I. M. , A Study on Some Reproductive Disorders in Dromedary Camel Herds in Saudi Arabia With Special References to Uterine Infections and Abortion, Tropical Animal Health and Production. (2017) 49, no. 5, 967–974, 10.1007/s11250-017-1284-x. 28364266

[64] Obaidat M. M. and Kersh G. J. , Prevalence and Risk Factors of *Coxiella burnetii* Antibodies in Bulk Milk From Cattle, Sheep, and Goats in Jordan, Journal of Food Protection. (2017) 80, no. 4, 561–566, 10.4315/0362-028X.JFP-16-377. 28272921 PMC6489127

[65] Alhetheel A. , Binkhamis K. , Somily A. , Barry M. , and Shakoor Z. , Screening for Q Fever: A Tertiary Care Hospital-Based Experience in Central Saudi Arabia, Saudi Medical Journal. (2018) 39, no. 12, 1195–1199, 10.15537/smj.2018.12.23695. 30520500 PMC6344652

[66] A Jarelnabi A. , Alshaikh M. A. , and Bakhiet A. O. , et al.Seroprevalence of q Fever in Farm Animals in saudi arabia, Biomedical Research. (2018) 29, no. 5, 895–900, 10.4066/biomedicalresearch.29-17-770.

[67] Alzahrani A. , Alqarni T. , Alsalmi M. , Ashi A. , and Waggass R. , Q Fever Endocarditis in a Saudi Child: A Case Report and Literature Review, Cureus. (2019) 11, no. 12, 10.7759/cureus.6322, e6322. 31938613 PMC6946032

[68] Elzein F. E. , Alsherbeeni N. , and Alnajashi K. , et al.Ten-Year Experience of Q Fever Endocarditis in a Tertiary Cardiac Center in Saudi Arabia, International Journal of Infectious Diseases. (2019) 88, 21–26, 10.1016/j.ijid.2019.07.035. 31382048

[69] Obaidat M. M. , Malania L. , Imnadze P. , Roess A. A. , Salman A. E. B. , and Arner R. J. , Seroprevalence and Risk Factors for *coxiella burnetii* in Jordan, The American Journal of Tropical Medicine and Hygiene. (2019) 101, no. 1, 40–44, 10.4269/ajtmh.19-0049. 31115294 PMC6609193

[70] Al-Araimi H. A. , Al-Alawi K. , and Al-Jardani A. K. , et al.Chronic Q Fever Endocarditis in an Omani Child: The First Pediatric Case Report From Oman, Oman Medical Journal. (2020) 35, no. 5, 10.5001/omj.2020.121, e180. 33083038 PMC7548047

[71] Alabdely M. H. , Mukhtar N. , and Alshaikh A. , et al.Q-Fever Prosthetic Valve Endocarditis in a Patient With SLE and Antiphospholipid Antibody Syndrome, Journal of Infection and Public Health. (2020) 13, no. 5, 821–823, 10.1016/j.jiph.2020.02.036. 32241725

[72] Barigye R. , Hassan N. A. , AlQubaisi D. M. N. , and Abdalla-Alfaki I. M. , Serological Evidence of *Coxiella burnetii*, *Leptospira interrogans* Hardjo, Neospora Caninum and Bovine Pestivirus Infections in a Dairy Cattle Herd From the United Arab Emirates, Veterinaria Italiana. (2020) 56, no. 3, 163–168. 33543911 10.12834/VetIt.2257.12932.1

[73] Gharban H. A. J. and Yousif A. A. , Serological and Molecular Phylogenetic Detection of *Coxiella burnetii* in Lactating Cows, Iraq, The Iraqi Journal of Veterinary Medicine. (2020) 44, no. (E0), 42–50, 10.30539/ijvm.v44i(E0).1020.

[74] Gharban H. A. J. and Yousif A. A. , Serological, Clinical and Hematological Prevalence of *Coxiella burnetii* in Adult Cows, Iraq, Biochemical and Cellular Archives. (2020) 20, no. 1, 67–74.

[75] Hardi F. M. , Rauf H. S. , Mahmood S. L. , Ahmad R. B. , Ali B. A. , and Sheikh M. O. B. , Molecular Detection and Identification of *Coxiella burnetii* in Aborted Sheep and Goats in Sulaimani Province, Kurdistan-Iraq, Assiut Veterinary Medical Journal (Egypt). (2020) 66, no. 164, 133–139, 10.21608/avmj.2020.167268.

[76] Lafi S. Q. , Talafha A. Q. , Abu-Dalbouh M. A. , Hailat R. S. , and Khalifeh M. S. , Seroprevalence and Associated Risk Factors of *Coxiella burnetii* (Q Fever) in Goats and Sheep in Northern Jordan, Tropical Animal Health and Production. (2020) 52, no. 4, 1553–1559, 10.1007/s11250-019-02153-0. 31820305

[77] Al-Ahmed T. A. and Ahmed T. H. , Molecular Confirmation of *Coxiella burnetii* in Naturally Infected Adult Cattle, Biochemical and Cellular Archives. (2021) 21, no. 1, 797–801.

[78] Al-Bayati L. H. , Razooqi M. A. , and Saleem H. D. , Serological Detection of *Coxiella burnetii* in Raw Milk of Goats in Baghdad, Iraq, Biochemical and Cellular Archives. (2021) 21, no. 2, 4071–4077.

[79] Al-Farwachi M. I. and Al-Robaiee I. A. , Seroprevalence Of Q Fever Among Sheep In Mosul City, Iraq, Archives of Veterinary Science. (2021) 26, no. 3, 82–87, 10.5380/avs.v26i3.80227.

[80] Al-Graibawi M. A. , Yousif A. A. , Gharban H. A. , and Zinsstag J. , First Serodetection and Molecular Phylogenetic Documentation of *Coxiella burnetii* Isolates From Female Camels in Wasit Governorate, Iraq, Iraqi Journal of Veterinary Sciences. (2021) 35, 47–52, 10.33899/ijvs.2021.130888.1890.

[81] Alqumber M. A. A. , Microbial Diversity of Molasses Containing Tobacco (Maassel) Unveils Contamination With Many Human Pathogens, European Review for Medical and Pharmacological Sciences. (2021) 25, no. 15, 4919–4929, 10.26355/eurrev_202108_26449. 34355364

[82] Barigye R. , Hassan N. A. D. , Abdalla Alfaki I. M. , Barongo M. B. , Mohamed M. E. H. , and Mohteshamuddin K. , Seroprevalence of *Coxiella burnetii* in a Dairy Cattle Herd From the Al Ain Region, United Arab Emirates, Tropical Animal Health and Production. (2021) 53, no. 1, 10.1007/s11250-021-02570-0. 33432436

[83] Barry M. , Bari S. A. , and Akhtar M. Y. , et al.Clinical and Microbiological Characteristics of Infective Endocarditis at a Cardiac Center in Saudi Arabia, Journal of Epidemiology and Global Health. (2021) 11, no. 4, 435–443, 10.1007/s44197-021-00013-5. 34735715 PMC8664328

[84] Elsohaby I. , Elmoslemany A. , and El-Sharnouby M. , et al.Flock Management Risk Factors Associated With Q Fever Infection in Sheep in Saudi Arabia, Animals. (2021) 11, no. 7, 10.3390/ani11071948, 1948. 34208803 PMC8300262

[85] Gharban H. A. J. and Yousif A. A. , First Isolation and Molecular Phylogenetic Analysis of *Coxiella burnetii* in Lactating Cows, Iraq, Bulgarian Journal of Veterinary Medicine. (2021) 24, no. 4, 508–519, 10.15547/bjvm.2322.

[86] Tigani-asil E. T. A. E. , Blanda V. , and Abdelwahab G. E. , et al.Molecular Investigation on Tick-Borne Hemoparasites and *Coxiella burnetii* in Dromedary Camels (*Camelus dromedarius*) in Al Dhafra Region of Abu Dhabi, UAE, Animals. (2021) 11, no. 3, 1–12. 10.3390/ani11030666 PMC8000914 33801532

[87] Al-Kindi N. , Al-Yaaqoubi M. , Al-Rashdi Y. , Al-Rashdi A. , Al-Ajmi A. , and Al-Maani A. , The First Confirmed Pediatric Chronic Osteomyelitis Due to *Coxiella Burnetii* in Oman, Oman Medical Journal. (2022) 37, no. 6, 10.5001/omj.2023.03, e449. 36458245 PMC9644042

[88] Alotaibi B. H. , Amor N. , Merella P. , Mohammed O. B. , and Alagaili A. N. , Correction to: Genetic Diversity of Wild Rodents and Detection of *Coxiella burnetii*, the Causative Agent of Q Fever, in Saudi Arabia, Veterinary Research Communications. (2022) 46, no. 3, 987–987, 10.1007/s11259-022-09903-w. 35179678

[89] Islam M. M. , Farag E. , and Hassan M. M. , et al.Diversity of Bacterial Pathogens and Their Antimicrobial Resistance Profile Among Commensal Rodents in Qatar, Veterinary Research Communications. (2022) 46, no. 2, 487–498, 10.1007/s11259-021-09876-2. 35083655

[90] Shati A. A. , Al-Taee H. S. R. , and Saleem H. D. , Ser-Surveying of Caprine Q-Fever (*Coxiella burnetii*) in Milk, Revista Electronica De Veterinaria. (2022) 23, no. 3, 467–478.

[91] Holloway P. , Gibson M. , and Nash S. , et al.A Cross-Sectional Study of Q Fever in Camels: Risk Factors for Infection, the Role of Small Ruminants and Public Health Implications for Desert-Dwelling Pastoral Communities, Zoonoses and Public Health. (2023) 70, no. 3, 238–247, 10.1111/zph.13019. 36601879 PMC10952281

[92] Alanbaki N. , Abdullah B. , Abbas W. , and Elbahnasawy M. , The Incidence of Coxiella Infection in Iraqi Women With Early Pregnancy Loss, Al Mustansiriyah Journal of Pharmaceutical Sciences. (2024) 24, no. 2, 150–162, 10.32947/ajps.v24i2.1032.

[93] Alkenani N. A. , Baroom H. M. , and Almohimeed A. A. , et al.Serological Investigation of *Coxiella burnetii* Infection (Query Fever) in Livestock in Makkah Province, Saudi Arabia, Veterinary World. (2024) 17, no. 4, 842–847, 10.14202/vetworld.2024.842-847. 38798290 PMC11111712

[94] Baroom H. M. , Alkenani N. A. , and Al-Johny B. O. , et al.Molecular Detection of *Coxiella burnetii* Infection (Q Fever) in Livestock in Makkah Province, Saudi Arabia, Zeitschrift fur Naturforschung. C, Journal of biosciences. (2024) 80, no. 5-6, 275–284, 10.1515/znc-2024-0126. 39438143

[95] Kadhim H. M. , Al-Hassani M. K. A. , Al-Galebi A. A. S. , and Essa I. M. , Serological and Molecular Prevalences and Phylogenetic Analysis of *Coxiella burnetii* in Dogs in Al-Qadisiyah and Baghdad Provinces, Iraq, Veterinary World. (2024) 17, no. 11, 2603–2611, 10.14202/vetworld.2024.2603-2611. 39829647 PMC11736363

[96] ElObeidy A. , Scientific System in the Arab Region: From Prestige Towards Development, Regional Science Policy & Practice. (2013) 5, no. 1, 97–113, 10.1111/j.1757-7802.2012.01089.x.

[97] Saaida M. , Problems of Scientific Research in the Arab World, International Journal of Research. (2021) 8, 100–105.

[98] Lippi M. , Sebastiani A. , and el-Mutabakani H. , Detection of Serum Antibodies Against Reoviruses, Adenoviruses and *Coxiella burneti* in a Group of Inhabitants of Riyad (Saudi Arabia), Archivio Italiano di Scienze Mediche Tropicali e di Parassitologia. (1968) 49, no. 5, 129–136. 4304504

[99] Ahmadinezhad M. , Mounesan L. , Doosti-Irani A. , and Behzadi M. Y. , The Prevalence of Q Fever in the Eastern Mediterranean Region: A Systematic Review and Meta-Analysis, Epidemiology and Health. (2022) 44, 10.4178/epih.e2022097, e2022097. 36317399 PMC10396516

[100] Nokhodian Z. , Feizi A. , Ataei B. , Hoseini S. G. , and Mostafavi E. , Epidemiology of Q Fever in Iran: A Systematic Review and Meta-Analysis for Estimating Serological and Molecular Prevalence, Journal of Research in Medical Sciences. (2017) 22, no. 1, 10.4103/jrms.JRMS_586_17. PMC5721492 29259632

[101] Gidding H. F. , Peng C. Q. , and Graves S. , et al.Q Fever Seroprevalence in Australia Suggests One in Twenty People Have Been Exposed, Epidemiology and Infection. (2020) 148, 10.1017/S0950268820000084, e18. 32019623 PMC7019564

[102] Islam M. M. , Dutta P. , and Bansal D. , et al.Prevalence and Risk Factors of Coxiellosis at the Human–Animal–Environment Interface in the South Asian Countries: A Systematic Review and Meta-Analysis, Transboundary and Emerging Diseases. (2025) 2025, no. 1, 10.1155/tbed/2890693, 2890693. 40302760 PMC12016896

[103] Abdally M. , Al-Marri T. , Hm A. , and Oa A.-J. , Incidence and Prevalence of Hard Ticks in Ruminants of Al-Ahsa Oasis Region, Kingdom of Saudi Arabia, Journal of World’s Poultry Research. (2020) 10, no. 3, 276–285, 10.36380/scil.2020.wvj36.

[104] Van den Brom R. , van Engelen E. , Roest H. I. , van der Hoek W. , and Vellema P. , *Coxiella burnetii* Infections in Sheep or Goats: An Opinionated Review, Veterinary Microbiology. (2015) 181, no. 1-2, 119–129, 10.1016/j.vetmic.2015.07.011. 26315774

[105] Cruz R. , Esteves F. , and Vasconcelos-Nóbrega C. , et al.Outbreaks of Abortions by *Coxiella burnetii* in Small Ruminant Flocks and a Longitudinal Serological Approach on Archived Bulk Tank Milk Suggest Q Fever Emergence in Central Portugal, Transboundary and Emerging Diseases. (2018) 65, no. 4, 972–975, 10.1111/tbed.12913. 29799172

[106] Pires H. , Santos-Silva S. , and Cruz A. V. S. , et al.Molecular Evidence of Sporadic *Coxiella burnetii* Excretion in Sheep Milk, Central Portugal, Veterinary Research Communications. (2024) 48, no. 4, 2713–2719, 10.1007/s11259-024-10389-x. 38656656 PMC11315700

[107] Farag E. , Sikkema R. S. , and Vinks T. , et al.Drivers of MERS-CoV Emergence in Qatar, Viruses. (2018) 11, no. 1, 10.3390/v11010022. PMC6356962 30602691

[108] Islam M. M. , Farag E. , and Hassan M. M. , et al.Rodent-Borne Zoonoses in Qatar: A Possible One-Health Framework for the Intervention of Future Epidemic, One Health. (2023) 16, 10.1016/j.onehlt.2023.100517, 100517. 37363248 PMC10288060

[109] Fair J. , Jentes E. , and Inapogui A. , et al.Lassa Virus-Infected Rodents in Refugee Camps in Guinea: A Looming Threat to Public Health in a Politically Unstable Region, Vector-Borne and Zoonotic Diseases. (2007) 7, no. 2, 167–171, 10.1089/vbz.2006.0581. 17627434

[110] Faulde M. , Rats and Mice-Neglected Vectors and Reservoirs of Dangerous Infectious Diseases?, Hygiene + Medizin. (2004) 29, 206–216.

[111] Farag E. , Nour M. , and Islam M. M. , et al.Qatar Experience on One Health Approach for Middle-East Respiratory Syndrome Coronavirus, 2012-2017: A Viewpoint, One Health. (2019) 7, 10.1016/j.onehlt.2019.100090, 100090. 31011617 PMC6462540

